# Association between Weight Status and Physical Fitness in Chinese Mainland Children and Adolescents: A Cross-Sectional Study

**DOI:** 10.3390/ijerph17072468

**Published:** 2020-04-04

**Authors:** Yatao Xu, Maorong Mei, Hui Wang, Qingwei Yan, Gang He

**Affiliations:** 1 Department of physical education & Health, Nanjing University of Finance & Economics, Nanjing 210046, China; xytao921@163.com (Y.X.); 9119931012@nufe.edu.cn (M.M.); 2 Key Laboratory of Adolescent Health Assessment and Exercise Intervention, Ministry of Education, College of Physical Education & Health, East China Normal University, Shanghai 200241, China; 3 Department of Physical Education, Xiamen University TKK College, Zhangzhou 363105, China; monkeykinghui@163.com; 4 College of Physical Education, Xizang Minzu University, Xianyang 712082, China; yan2004@126.com; 5 College of SWAT, Nanjing Forest Police College, Nanjing 210046, China

**Keywords:** physical fitness, adolescents, underweight, obese, Chinese mainland

## Abstract

*Background*: The increasing prevalence of obesity among children and adolescents is a major public health challenge worldwide. This study examined the relationship between physical fitness and BMI spanning the range from underweight to obese among Chinese mainland children and adolescents. *Methods*: Participants were 22,681 children and adolescents (11,300 boys and 11,381 girls) aged 10–18 years from the Chinese mainland. Weight status was classified as underweight, normal weight, overweight, and obese using WHO 2007 standards. Physical fitness parameters such as cardiorespiratory fitness (VO_2max_), lower body explosive strength (standing broad jump), upper body explosive strength (handgrip strength), abdominal muscular endurance (sit-ups in 30 s), flexibility (sit-and-reach), and agility (repeat bestride (20 s)) were assessed. *Results*: There was a significant association between weight status categories and physical fitness in all age groups and sex (*p*_linear_ < 0.001, *p*_quadratic_ < 0.001). Underweight adolescents performed better in lower limb strength, flexibility, agility, and cardiorespiratory fitness than their obese peers, but worse in upper limb strength. Underweight boys aged 10–11 and 12–13 years and girls aged 10–11 years showed significantly (*p* < 0.05) high odds of meeting a low physical fitness index. Obese adolescents have high odds of meeting a low physical fitness index with age. *Conclusion*: The present study showed a nonlinear relationship between weight status and physical fitness. Children and adolescents who were classified as underweight or obese had poorer physical fitness than their normal-weight peers.

## 1. Introduction

The prevalence of obesity in children and adolescents is one of the most serious threats to public health in the 21st century, worldwide [1]. Obesity in childhood is associated with poor health in adulthood [2]. Physical fitness is an important marker of health and growth in children and adolescents [3]. Physical fitness is also a predictor of suffering chronic diseases, such as obesity, cardiovascular disease, and mental health [4].

The prevalence of obesity is rapidly increasing among Chinese children and adolescents [5]. Therefore, it is important to explore the relationship between high body mass index (BMI) and physical fitness. Previous studies found that BMI had a negative influence on fitness test performance, especially in the parameters requiring quick movements and body displacement [6,7,8]. Few studies reported a linear relationship between BMI and fitness [9,10], whereas others found a nonlinear relationship among them [8,11]. Generally, previous studies researched the relationship between excess body weight and physical fitness. It is possible that at the other extreme, a low BMI might have a negative influence on some measures of physical fitness [6]. There are a number of underweight children and adolescents worldwide, especially in some developing countries in Latin America, Africa, and Asia [12]. In 2014, the prevalence of malnutrition among Chinese Han students aged 7–18 years was 10.0%. The prevalence of severe and mild wasting was 3.7% and 5.5%, respectively [13].

In China, most studies focus on the adverse effects of high BMI on physical fitness but ignore the potential impact of low BMI on physical fitness. Only a few studies in Hong Kong [14] and Taiwan [6,7] have found a potential nonlinear relationship between them and have noted that underweight adolescents scored better than their overweight/obese peers [15]. No research has examined the relationship between physical fitness and BMI spanning the range from underweight to obese in Chinese mainland adolescents. In this context, the purpose of this study was to evaluate the relationship between BMI categories (underweight, normal, overweight, and obese) and performance in physical fitness tests among Chinese mainland youth aged 10–18 years.

## 2. Materials and Methods

### 2.1. Participants

A sample of Chinese children and adolescents (10–18 years) was selected by means of a multiple-step, simple random sampling from primary school, middle school and high school from 26 provinces, and random assignment of the schools within each city. All of the randomly selected schools agreed to participate in this study during the PE classes. The study was approved by the Institutional Review Board of East China Normal University (HR 006-2019). Written informed consent was obtained from the adolescents involved in the study and their parents or guardians.

### 2.2. Anthropometric Measurements

Anthropometric measurements, including height and weight were assessed, while the subjects wore light clothing and no shoes. Height and weight tests were in accordance with the “National student physical health survey report 2010” [16] physique and health test conditions. BMI was calculated by dividing weight (kg) by height squared (m^2^). All measures were taken twice but not consecutively. The mean of the two measurements was used in the analyses. The BMI category of each person was classified as underweight (BMI < −2SD), normal weight (−2SD ≤ BMI < 1SD), overweight (1SD ≤ BMI < 2SD), and obese (BMI ≥ 2SD). The age and sex-specific cut-off values were as suggested by the WHO 2007 growth reference (5–19 years) [17].

### 2.3. Physical Fitness

Physical fitness was assessed by the following components: Cardiorespiratory fitness (VO_2max_), muscular strength (standing broad jump, handgrip strength), muscular endurance (sit-ups in 30 s test), flexibility (sit-and-reach) and agility (repeat bestride).

#### 2.3.1. Upper Body Explosive Strength

The handgrip strength test measures the upper body explosive strength using a calibrated and adjustable hand dynamometer (EH101; Zhongshan, China). Subjects were asked to hold it in their preferred hand from a standing position and squeeze it gradually and continuously up to the maximum for at least 2 s. The better of two attempts was considered the test score in kilograms.

#### 2.3.2. Lower Body Explosive Strength

The standing long jump test measures the lower limb power. Each participant did jumps for a distance from a standing start, arms forward, feet simultaneously off and landing on the ground, trying to jump as far as possible. The better of two attempts was the score, in centimeters.

#### 2.3.3. Abdominal Muscular Endurance

The sit-ups (30 s) test measures abdominal muscular endurance. First, subjects lay on a carpeted or cushioned floor with arms crossed on the chest, with knees bent at approximately right angles and feet flat on the ground. Second, their upper body was to lift with their elbows out in front to touch or go beyond their knees and then return to the lying position. The total number of correctly performed complete sit-ups in 30 s was the score.

#### 2.3.4. Flexibility

For the sit-and-reach assessment of flexibility, the subjects sat on the flat ground with back, shoulders and the back of the head close to the wall, arms were straightened and hands were put on the test instrument board with the palms facing down and legs straight. At the beginning of the test part, the upper body bends forward to push the test instrument slowly until it could not be pushed further.

#### 2.3.5. Agility

For the repeat bestride assessment (agility), the subjects were required to stand with both feet on the centerline of a long board with parallel lines of 100 cm on both sides. The subjects were required to go over the line on the right, return to the centerline, and then go over the line on the left, and return to the original position on the center line which was recorded once. One repetition was recorded each time the subject crossed the line, and the total number of repetitions within 20 s was recorded.

#### 2.3.6. Cardiorespiratory Fitness

Participants had to run back and forth between two lines 20 m apart with the music signal. The test finished when the child failed to reach the end lines concurrent with the music signal 2 times in succession or when the individual stops because of exhaustion. The rhythm of the music was increased by one level every 1 min, the initial speed was 8.5 km/h, and the speed of each additional level increased by 0.5 km/h.

VO_2max_ was according to formula as follows:

VO_2max_ = 61.1 − 2.20×gender − 0.462 × age − 0.862 × BMI + 0.192 × reps [18]

Note: boys = 0, girls = 1

### 2.4. Statistical Analyses

Descriptive statistics for all variables were calculated separately for girls and boys by age groups 10–11, 12–13,14–15, and 16–18 years. χ²-test was used for sexual weight status. ANCOVA was performed to compare the physical fitness of BMI categories as underweight, normal weight, overweight, and obese. The method also examined both linear and quadratic trends.

The physical fitness indicators of different sex and ages were standardized and the sum of Z scores of each physical fitness indicator to obtain the physical fitness index was calculated. The physical fitness index was divided into two groups: high physical fitness and low physical fitness according to the P_75_ percentile cutoffs [19]. Logistic regression models were estimated to match the physical fitness index and weight status categories with gender and age, using normal weight and low physical fitness as reference. Analysis was performed using SPSS 23.0 (IBM Corporation, Armonk, NY, USA). The level of significance was set at *p* < 0.05.

## 3. Results

Table 1 shows that the percentage of underweight boys was 3.4%, whereas underweight girls were 4.5% for adolescents of 10–11 years, while the percentage was higher than girls in other age groups (*p* < 0.001). The percentage of overweight and obesity in boys was significantly (*p* < 0.001) higher than girls in all age groups.

Table 2 shows that the physical fitness tests of handgrip strength, standing board jump, sit-ups 30 s test, repeat bestride 20 s test, and VO_2max_ were performed significantly better by the boys compared to the girls in each age group (*p* < 0.001). Girls performed better than the boys in each age group in the sit-and-reach test (*p* < 0.05).

**Figure 1:**
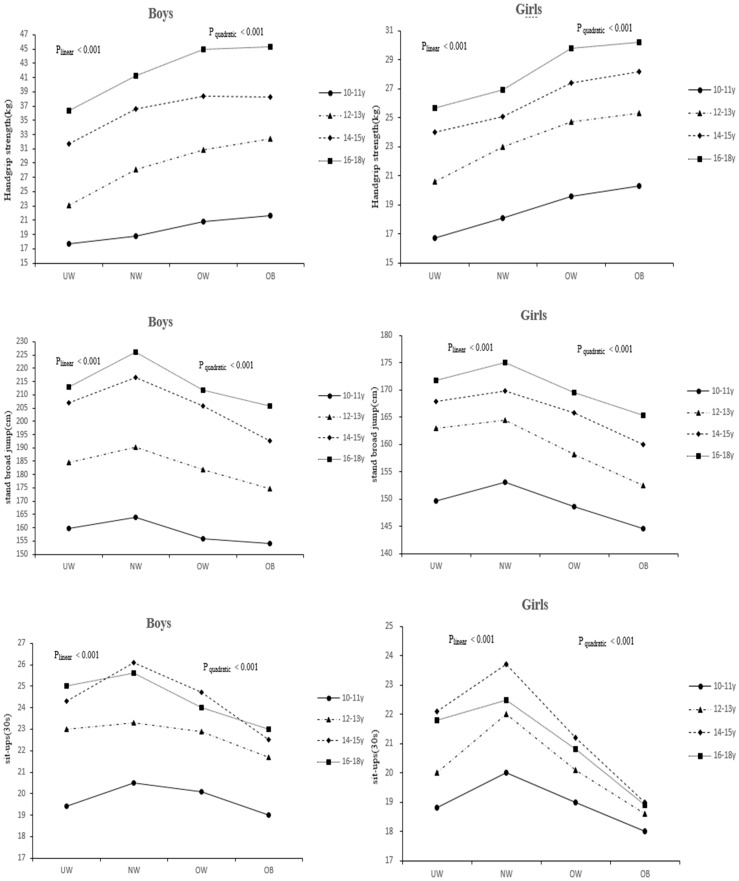

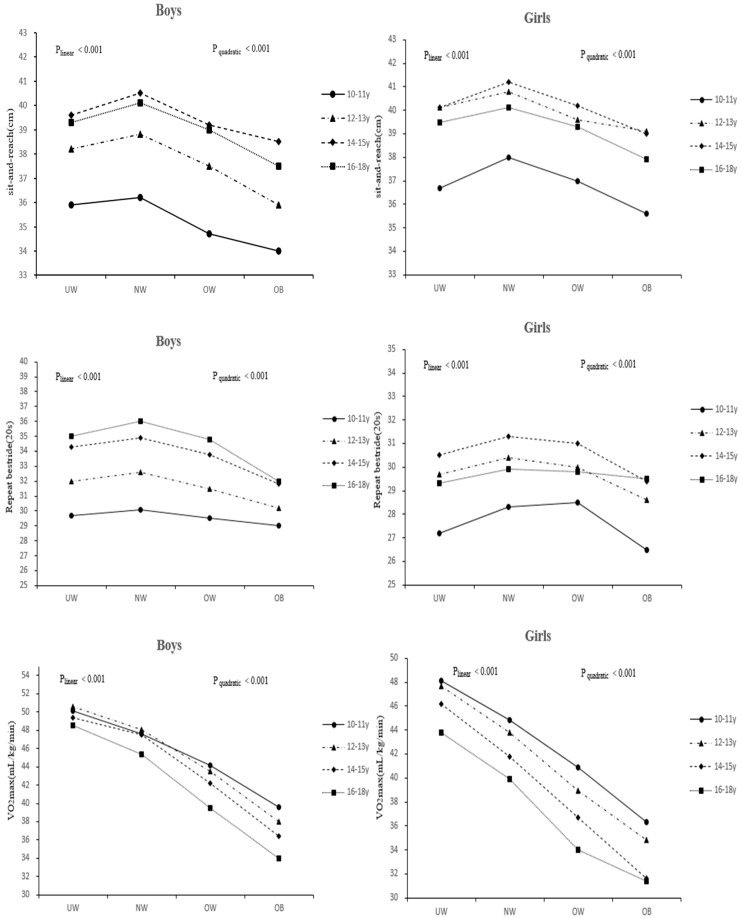
Association of physical fitness in boys and girls across BMI categories in adolescents. *p*_linear_ and *p*_quadratic_ refer to *p*-values obtained from ANCOVA analysis for linear and quadratic term. UW = underweight; NW = normal weight; OW = overweight; OB = obese.

Results of the linear and quadratic trend analysis showed a significant association between weight status categories and physical fitness in all age groups and genders (*p*_linear_ < 0.001, *p*_quadratic_ < 0.001), see Figure 1.

When adjusted for region and family income (monthly), the results showed that normal-weight groups showed a better performance than the other BMI categories in the standing board jump, sit-ups 30 s test, sit-and-reach, and repeat bestride. Children and adolescents who were classified as obese performed better in the hand strength than their peers among the four weight status groups.

VO_2max_ was used to estimate the cardiorespiratory fitness of individuals. There was a well-defined linear relationship between BMI and VO_2max_. Underweight adolescents performed better than their peers of VO_2max_. Obese children performed the worst among the four weight status groups (*p* < 0.001).

Table 3 presents the results of the multi-level logistic regression analysis describing the association between the weight status and physical fitness index. The results showed that underweight boys and girls aged 10–11years (boys OR = 1.579; girls OR = 1.579, *p* < 0.05) had higher odds to meet a lower physical fitness index compared with their normal-weight peers. In addition, obese children and adolescents aged 12–18 years are more likely to meet a low physical fitness index.

## 4. Discussion

The aim of the present study was to explore the association between BMI categories (underweight, normal, overweight, and obese) and physical fitness among Chinese mainland children and adolescents. The main finding of this study was that BMI and physical fitness have a significant relationship.

The present results showed that the handgrip strength was increased with weight growth in both genders. This may be due to lower fat-free mass in underweight children and higher fat-free mass in obese children. These results are in accordance with a previous study by Silverman et al. [20] who were researched the trend of handgrip strength of children in the United States and Canada. It was found that there is a positive correlation between body mass and handgrip strength. Ervin et al. [21] found that handgrip performance in obese children is better than underweight children.

The association between weight status and physical fitness varies with the type of test performed. In the present study, obese boys and girls showed a poorer standing broad jump and repeat bestride results than the other three BMI category peers. A previous study suggested that a higher weight was a barrier in fitness tests that required quick position changes because body weight increased the forces exerted on the knee extension [10] thus, leading to difficulty for children to conduct tests involving moving their weight or keeping it in the right position.

Previous research found that excess body weight restricted activities during excise leading to decreased muscular endurance when compared to a normal-weight person [22]. Our research showed that obese adolescents performed worst in sit-ups compared to other weight status groups in both genders. This was due to higher fat and lower muscle mass in the waist area. Another study suggested that excessive fat limited the ability of a continuous supply of oxygen to muscle mass [6]. The reason for poor sit-ups performance in underweight individuals is unknown.

The results showed that obese children and adolescents had poorer flexibility compared with other individuals, which is in agreement with a previous study [23]. Casonatto et al. [24] investigated the association between BMI status and physical performance in Brazilian children and found that abdominal obesity might affect the lower back and hamstring flexibility and hamper the trunk to the extreme reach position. But another study found that a higher BMI was strongly associated with performances in fitness tests except flexibility, in Greek children [25]. In addition, underweight adolescents showed poorer sit-and-reach performance than normal-weight youth. The reason may result from inhibition of flexibility by early onset of puberty. Ruiz et al. [4] suggested that it would be interesting to investigate if weaker abdominal strength might affect the ability of underweight subjects. Future research needs more attention to the relation between underweight and flexibility.

The present study showed that obese individuals had lower cardiorespiratory fitness which is in accordance with the previous study [26]. Underweight children and adolescents performed superior to obese individuals between four BMI categories because the lower weight of underweight adolescents is helpful in endurance running. The biological mechanism of obesity has shown that overweight and excess body fat reduces an individual’s exercise tolerance and aerobic capacity compared to normal-weight children [7]. Addtionally, being overweight and obese made the children lose their self-confidence and motivation to participate in physical exercise, resulting in a poorer performance in physical fitness than their peers.

The previous study has found that the relationship between BMI and physical fitness varies with gender and maturity status level [8]. The results in the present study showed that underweight boys and girls aged 10–11years had a higher risk (boys OR = 1.579; girls OR = 1.579 *p* < 0.05) of meeting a lower physical fitness index than normal-weight peers. The reason may be because a poor nutritional status is considered to have a negative effect on activity which further leads to lower physical fitness [27]. In the middle and late stages of puberty, various functions and organ development tend to be stable. Therefore, the negative effects of excessive fat accumulation on body weight gradually emerges.

There are limitations to the present study. Firstly, many factors affected physical fitness, thus a cross-sectional method could not reflect the link between BMI and physical fitness. Secondly, the present study used some new methods which are different from the “National physical fitness test”, thus the proficiency of methods would affect the results, which may not truly reflect the actual physical fitness level. Thirdly, we did not research the association between abdominal obesity and physical fitness. The strength of this study is that the large sample size allowed us to explore the association between different weight status and physical fitness among children and adolescents from the Chinese mainland.

## 5. Conclusions

In conclusion, the present study showed a generally nonlinear relationship between weight status and physical fitness. Children and adolescents who were classified as underweight or obese had poorer physical fitness than their normal-weight peers. Results also suggested that obese children and adolescents generally had higher odds of meeting a low physical fitness index when compared to their normal-weight peers.

## Figures and Tables

**Table 1:** Prevalence of underweight, normal weight, overweight, and obesity.

Gender/Years	Underweight N (%)	Normal Weight N (%)	Over Weight N (%)	Obesity N (%)	*p*-Value
10–11					
Boys	84 (3.4)	1335 (54.1)	595 (24.1)	453 (18.4)	0
Girls	115 (4.5)	1786 (69.7)	497(19.4)	166(6.5)	
12–13					
Boys	86 (3.4)	1536 (61.2)	565 (22.5)	323 (12.9)	0
Girls	64 (2.6)	1929 (77.3)	394 (15.8)	108 (4.3)	
14–15					
Boys	71 (2.8)	1792 (71.5)	434 (17.3)	209 (8.3)	0
Girls	56 (2.2)	2111 (84.6)	283 (11.3)	46 (1.8)	
16–18					
Boys	134 (3.5)	3048 (79.9)	461 (12.1)	174 (4.6)	0
Girls	112 (2.9)	3497 (91.4)	199 (5.2)	18 (0.5)	

χ²-test for weight status for gender, *p* < 0.001 means all weight status have a significant difference between both age group and gender.

**Table 2:** Physical fitness tests, means, and standard deviations for sex and age group.

Gender/Years	N	Hand Strength	Standing Board Jump	Sit-Ups	Sit-and-Reach	Repeat Bestride	VO_2max_	*p*-Value
Boys		M (SD)	M (SD)	M (SD)	M (SD)	M (SD)	M (SD)	
10–11	2467	19.8 (5.8)	161.1 (23.7)	20.1 (5.9)	35.3 (10.9)	29.5 (8.2)	45.4 (4.2)	
12–13	2510	29.1 (10.5)	186.2 (27.9)	23.0 (6.3)	37.7 (10.7)	32.3 (9.2)	45.9 (5.1)	
14–15	2506	36.9 (8.9)	212.3 (28.1)	25.5 (7.0)	39.3 (11.1)	34.3 (9.1)	45.7 (5.2)	
16–18	3817	41.7 (8.5)	223.1 (26.4)	25.3 (6.7)	39.4 (11.4)	34.5 (9.5)	44.3 (5.0)	
Girls								
10–11	2564	18.5 (6.2)	151.5 (21.6)	19.3 (5.8)	37.1 (11.0)	28.2 (7.7)	43.6 (3.6)	0.000
12–13	2495	23.3 (5.4)	162.2 (20.7)	20.0 (5.7)	40.0 (10.9)	30.2 (9.9)	42.8 (4.0)	0.000
14–15	3496	25.4 (7.1)	169.0 (20.3)	21.6 (6.5)	40.1 (10.7)	30.4 (7.8)	41.1 (3.7)	0.000
16–18	3826	27.0 (8.2)	168.9 (19.0)	21.4 (6.0)	40.2 (11.0)	29.9 (8.2)	39.7 (3.5)	0.000

M = mean; SD = standard deviation; hand strength (kg); standing board jump(cm); sit-ups 30 s test (reps); sit-and-reach (cm); repeat bestride 20s test (reps); VO_2max_, maximal oxygen consumption (mL/(kg·min).

**Table 3:** Multi-level regression for physical fitness index and weight status by sex and age.

Age/Weight Status	Boys			Girls		
	OR	95 CI	*p*-Value	OR	95 CI	*p*-Value
10–11years						
NW	1.00			1.00		
UW	1.309	0.758–2.263	0.034	1.579	0.972–2.566	0.043
OW	0.747	0.603–0.926	0.008	0.887	0.710–1.108	0.290
OB	0.957	0.749–1.223	0.728	1.382	0.930–2.054	0.110
12–13years						
NW	1.00			1.00		
UW	2.049	1.123–3.739	0.019	1.445	0.764–2.732	0.257
OW	1.039	0.833–1.295	0.735	1.094	0.846–1.416	0.493
OB	1.644	1.212–2.229	0.001	1.912	1.113–3.285	0.019
14–15years						
NW	1.00			1.00		
UW	2.900	1.883–4.466	0.003	0.785	0.434–1.422	0.425
OW	1.516	1.175–1.957	0.001	1.084	0.804–1.461	0.597
OB	3.089	1.466–5.509	0.000	1.459	0.675–3.153	0.036
16–18years						
NW	1.00			1.00		
UW	1.580	1.019–2.451	0.041	0.354	0.139–0.900	0.838
OW	1.542	1.205–1.974	0.001	0.955	0.613–1.487	0.764
OB	3.512	2.109–5.846	0.000	1.055	0.745–1.934	0.029

Reference group: normal weight adolescents, high physical fitness index; adjusted by region, nation, and socio-economic status. UW = underweight; NW = normal weight; OW = overweight; OB = obese.
